# *Streptomyces harenosi* sp. nov., a home for a gifted strain isolated from Indonesian sand dune soil

**DOI:** 10.1099/ijsem.0.004346

**Published:** 2020-08-21

**Authors:** Ali Budhi Kusuma, Imen Nouioui, Hans-Peter Klenk, Michael Goodfellow

**Affiliations:** ^1^​ School of Natural and Environmental Sciences, Ridley Building 2, Newcastle University, Newcastle upon Tyne NE1 7RU, UK; ^2^​ Indonesian Centre for Extremophile Bioresources and Biotechnology (ICEBB), Faculty of Biotechnology, Sumbawa University of Technology, Sumbawa Besar, 84371, Indonesia; ^3^​ Leibniz-Institut DSMZ – German Collection of Microorganisms and Cell Cultures, Inhoffenstraße 7B, 38124 Braunschweig, Germany

**Keywords:** *Streptomyces harenosi*sp. nov, arid sandy soil, polyphasic taxonomy, genomics

## Abstract

A polyphasic study was undertaken to establish the position of a *Streptomyces* strain, isolate PRKS01-65^T^, recovered from sand dune soil collected at Parangkusumo, Yogyakarta Province, Java, Indonesia. A combination of chemotaxonomic, cultural and morphological properties confirmed its position in the genus of *Streptomyces *. Comparative 16S rRNA gene sequence analyses showed that the isolate was most closely related to *Streptomyces leeuwenhoekii* C34^T^ (99.9 % similarity) and loosely associated with the type strains of *Streptomyces chiangmaiensis* (98.7 % similarity) and *Streptomyces glomeratus* (98.9 % similarity). Multilocus sequence analyses based on five conserved housekeeping gene alleles confirmed the close relationship between the isolate and *S. leeuwenhoekii* C34^T^, although both strains belonged to a well-supported clade that encompassed the type strains of *S. glomeratus *, *Streptomyces griseomycini *, *Streptomyces griseostramineus *, *Streptomyces labedae *, *Streptomyces lomondensis* and *Streptomyces spinoverrucosus *. A comparison of the draft genome sequence generated for the isolate with corresponding whole genome sequences of its closest phylogenomic neighbours showed that it formed a well-separated lineage with *S. leeuwenhoekii* C34^T^. These strains can also be distinguished using a combination of phenotypic properties and by average nucleotide identity and digital DNA–DNA hybridization similarities of 94.3 and 56 %, values consistent with their classification in different species. Based on this wealth of data it is proposed that isolate PRKS01-65^T^ (=NCIMB 15211^T^=CCMM B1302^T^=ICEBB-03^T^) be classified as *Streptomyces harenosi* sp. nov. The genome of the isolate contains several biosynthetic gene clusters with the potential to produce new natural products.

## Introduction

The emended family *Streptomycetaceae* [1, 2] includes the genus *Streptomyces* [1], the type genus [1] and five other genera. The genus *Streptomyces* encompasses over 800 species with validly published names (www.bacterio.net.streptomyces.html) most of which have been assigned to multi- and single-membered phyletic lines in *Streptomyces* 16S rRNA gene trees [3, 4]. Genera belonging to the family *Streptomycetaceae* can be distinguished using a combination of genomic, genotypic and phenotypic features [3, 4].

Streptomycetes are best known as a source of new antibiotics, anti-cancer and other specialized (secondary) metabolites [5, 6], hence the continued interest in them as a source of novel drugs needed in the fight against multi-drug resistant microbial pathogens [7, 8]. Previously unknown *Streptomyces* strains isolated from deep-sea sediments and desert soils, using taxonomic approaches to drug discovery [9, 10], are a rich source of new bioactive compounds [11, 12]. A case in point was the discovery of new natural products with anti-cancer and anti-microbial properties from *Streptomyces leeuwenhoekii* strains isolated from hyper-arid Atacama Desert soil [10, 13–16]. The type strain of this species, isolate C34^T^, is especially gifted *sensu* Baltz [17] as it has a large genome (7.8 Mb) that includes 31 biosynthetic gene clusters (BGCs) that mainly encode for unknown specialized metabolites [18]. Such gifted *Streptomyces* strains are pivotal in the search for new drug-leads using state-of-the-art technologies such as genome mining and metabolic engineering [19, 20].

The aim of the present study was to establish the taxonomic status of a *Streptomyces* strain, isolate PRKS01-65^T^, recovered from an arid Indonesian sandy soil sample and found to be closely related to the type strain of *S. leeuwenhoekii *. These strains were the subject of a polyphasic study underpinned by genomic data derived from whole genome sequences. The resultant datasets confirmed that the isolate and the *S. leeuwenhoekii* strain are closely related but belong to different species. Consequently, the isolate is considered to represent a new species of *Streptomyces* for which the name *Streptomyces harenosi* sp. nov. is proposed. The type strain, PRKS01-65^T^ (=NCIMB 15211^T^=CCMM B1302^T^=ICEBB-03^T^), is a potential source of new natural products.

## Isolation, maintenance and cultivation

Isolate PRKS01-65^T^ was recovered from an arid, non-saline soil sample (pH 5.8, organic matter content 0.06 %) collected just below the surface of a sand dune in the Parangkusumo region (8° 1’ 7.513″ S, 110° 19′ 11.04″ E) of Yogyakarta Province, Java, Indonesia by Ali Budhi Kusuma and his students in January 2013 (Fig. S1, available in the online version of this article). One gram of the soil sample was heated at 120 °C for 15 min, sprinkled directly over plates of actinomycete isolation agar (HiMedia), pH 7.3, that were incubated at 45 °C for up to 14 days [21]. Spores taken from a colony of isolate PRKS01-65^T^ growing on one of the isolation plates were used to inoculate yeast extract–malt extract agar [International *Streptomyces* Project (ISP) medium 2 [22]] plates., which were incubated at 28 °C for 7–14 days. Working cultures of the isolate and *S. leeuwenhoekii* C34^T^ [14] were maintained on ISP2 agar plates for long-term preservation the strains were kept as mixtures of hyphal fragments and spores in 20 %, v/v glycerol at −20 °C and −80 °C. Biomass for the chemotaxonomic analyses conducted on the isolate was harvested from ISP2 broth cultures which had been shaken at 180 r.p.m. in baffled flasks for 14 days at 28 °C following inoculation with 25 ml seed culture of the isolate prepared under the same conditions. The harvested biomass was washed twice in sterile distilled water and freeze-dried.

## Chemotaxonomic, cultural and morphological properties

Isolate PRKS01-65^T^ was examined for chemotaxonomic, cultural and morphological properties known to be of value in *Streptomyces* systematics [4, 23]. Isomers of diaminopimelic acid (A_2_pm) were sought as described by Staneck and Roberts [24], whole-organism sugars after Lechevalier and Lechevalier [25], and isoprenoid quinones and polar lipid profiles following the integrated procedure of Minnikin and his colleagues [26]. Fatty acids extracted from the isolate, cultivated under the same conditions as in an earlier study on *S. leeuwenhoekii* C34^T^ [14], were methylated and analysed using the Sherlock Microbial Identification (midi) system and the resultant peaks identified using the actino6 database [27]. Gram-stain and micromorphological properties were recorded following growth on ISP2 agar for 7 days at 28 °C. Growth taken from the ISP2 agar plate was examined for spore-chain arrangement and spore- surface ornamentation using a scanning electron microscope (Tescan Vega 3, LMU instrument) following the procedure described by O’Donnell *et al*. [28]. Cultural properties of the isolate were recorded from tryptone–yeast extract, yeast extract–malt extract, oatmeal, inorganic salts–starch, glycerol–asparagine, peptone–yeast extract–iron and tyrosine agar plates (ISP media 1–7) [22] after 21 days at 28 °C. Aerial and substrate mycelial colours and those of diffusible pigments were determined by comparisons against colour charts [29].

The chemotaxonomic, cultural and morphological properties of the isolate were consistent with its classification in the genus *Streptomyces* [30]. The organism was Gram-stain-positive, formed circular colonies bearing a grey aerial spore mass (Fig. S2a) and an extensively branched substrate mycelium carrying aerial hyphae that differentiated into spiral chains of smooth surfaced spores (Fig. S2b). Whole-organism hydrolysates of the strain were rich in ll-A_2_pm (Fig. S3), galactose and ribose with lesser proportions of glucose and mannose (Fig. S4). The major isoprenologs were hexa- and octa-hydrogenated menaquinones (34 and 66 %, respectively) and the polar lipids included diphosphatidylglycerol, phosphatidylethanolamine (diagnostic component), phosphatidylinositol and phosphatidylinositol mannosides (Fig. S5). The major fatty acids were in very good agreement with those found in *S. leeuwenhoekii* C34^T^ [14], apart from differences in minor components, as exemplified by the absence of iso-C_10 : 0_, C_14 : 0_ and C_17 : 0_ in the profile of isolate PRKS01-65^T^ (Table 1).

Table S1 shows that the isolate and *S. leeuwenhoekii* C34^T^ grew well on all of the ISP media. In general, the isolate formed a white to greyish-white aerial spore mass and a brown substrate mycelium on these media and the *S. leeuwenhoekii* strain an olivaceous greenish-grey aerial spore mass and a yellowish-white substrate mycelium. A brown diffusible pigment was produced by the isolate on ISP media 1, 2 and 4 whereas *S. leeuwenhoekii* C34^T^ exhibited yellowish to yellowish-grey diffusible pigments on all the ISP formulations, apart from ISP media 1 and 5.

**Table 1.** Cellular fatty acids (%) of isolate PRKS01-65^T^ and *Streptomyces leeuwenhoekii* C34^T^ –, Not detected; Data for the *S. leeuwenhoekii* strain was taken from Busarakam *et al*. [14].

Fatty acid	Isolate PRKS01-65^T^	* Streptomyces leeuwenhoekii* C34^T^
**Branched**		
antesio-C_15 : 0_	22.9	29.2
antesio-C_17 : 0_	10.2	13.8
antesio-C_17 : 1_ ω9*c*	5.1	1.1
iso-C_10 : 0_	1.1	–
iso-C_11 : 0_	2.0	–
iso*-*C_14 : 0_	7.4	4.3
iso-C_15 : 0_	4.6	5.5
iso-C_16 : 0_	36.6	12.5
iso*-*C_17 : 0_	2.8	4.4
iso*-*C_18 : 0_	–	0.9
iso-C_16 : 1_-H	2.8	–
**Saturated**		
C_14 : 0_	–	1.1
C_16 : 0_	2.9	19.1
C_17 : 0_	–	3.5
C_17 : 0_ cyclo	1.7	–

## Whole genome sequencing

Genomic DNA was extracted from wet biomass of a single colony of the isolate grown on ISP2 agar for 7 days at 28 °C following the protocol provided by MicrobesNG (Birmingham, UK; www.microbesng.uk) and sequenced using a Miseq instrument (Illumina). Genomic DNA libraries were prepared at MicrobesNG using a Nextera XT library preparation kit. The purity and concentration of the extracted genomic DNA was measured using the Microlab star liquid handling system (Hamilton) and libraries determined with the Kapa Biosystem library quantification kit designed for Illumina instruments on a LightCycler 96 real time PCR instrument (Roche). The libraries were sequenced following the 2×250 bp paired-end protocol (MicrobesNG). Reads were trimmed using Trimmomatic software version 0.38 [31] and their quality assessed with in-house scripts and SAMtools, BedTools and BWA-MEM software [32–34]. Reads under 200 bp were discarded and contigs assembled using SPAdes software version 3.1.1 [35]. The draft genome assembly was annotated using the rastweb server [36, 37] with default options and is available from GenBank (accession number WYCT00000000). The isolate was found to have a draft genome size of 8.0 Mb with 175× mean coverage, 1096 contigs and 66 tRNA genes, a single 16S rRNA gene, 7 23S rRNA genes, 7137 coding sequences (CDS) and an *in silico* G+C content of 73 mol%. Genomic features of the *S. leeuwenhoekii* have been reported [18].

## Phylogeny

An almost full-length 16S rRNA gene sequence (1528 bp; GenBank accession number MK503548) was extracted directly from the draft genome of the isolate using the ContEst16S tool available from the EZBioCloud webserver (www.ezbiocloud.net/tools/contest16s) [38]. The resultant 16S rRNA gene sequence was compared with corresponding sequences of the type strains of the most closely related *Streptomyces* species retrieved from the EzBiocloud server [39] following multiple sequence alignment generated using muscle software [40]. Pairwise sequence similarities were determined using the single-gene tree option in the Genome-to-Genome Distance Calculator (GGDC) web server [41, 42]. Phylogenetic trees were inferred using the maximum-likelihood [43], maximum-parsimony [44] and neighbour-joining [45] algorithms. The resultant trees were evaluated using bootstrap analyses based on 1000 replicates [46] from the mega X software package [47] using the two-parameter model of Jukes and Cantor [48]. The trees were rooted using a 16S rRNA gene sequence from *Streptomyces albus* subsp. *albus* NRRL B-1811^T^ (GenBank accession number JX486031.1), the type strain of the type species of the genus *Streptomyces *.

**Fig. 1.**
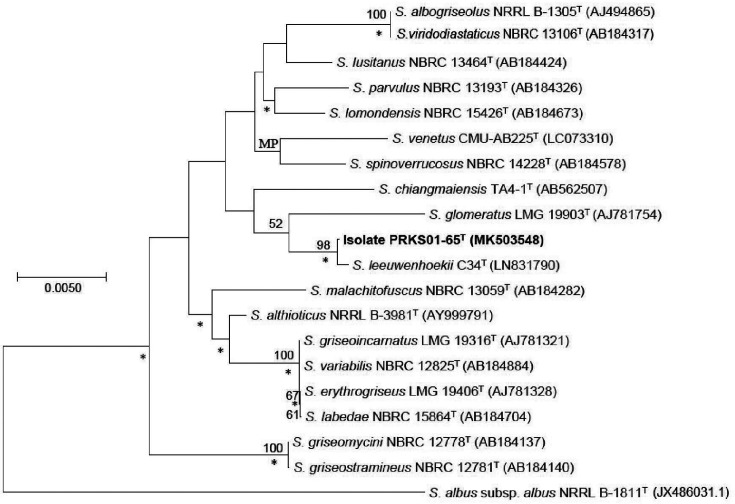
Neighbour-joining tree based on 16S rRNA gene sequences showing relationships between isolate PRKS01-65^T^ and the type strains of closely related *Streptomyces* species. Asterisks indicate branches of the tree that were found using the maximum-likelihood and maximum-parsimony algorithms. MP denote nodesrecovered using the maximum-parsimony tree-making algorithm. Numbers at the nodes indicate bootstrap values, only values above 50 % are shown. The root of the tree was established using *Streptomyces albus* subsp. *albus* NRRL B-1811^T^. Bar, 0.005 substitutions per nucleotide position.

The phylogenetic trees (Fig. 1) show that the isolate forms a well-supported branch in the *Streptomyces* 16S rRNA gene tree together with *S. leeuwenhoekii* C34^T^; the type strain of *Streptomyces glomeratus* [49] joins this group, albeit with low bootstrap support. *Streptomyces chiangmaiensis* TA4.1^T^ [50] is associated with this taxon but without statistical support. The isolate shares 16S rRNA gene sequence similarities with these strains of 99.9, 98.8 and 98.9 %, respectively. The last two values are well below the threshold recommended by Meier-Kolthoff *et al*. [42] for assigning closely related actinobacteria to the same species. The isolate shares sequence similarities of either 98.7 or 98.8 % with the remaining *Streptomyces* type strains. In general, these results correspond to those reported by Busarakam *et al*. [14], who noted that relationships between *S. leeuwenhoekii* C34^T^ and closely related *Streptomyces* species were sensitive to the treeing algorithms used, indicating that this part of the *Streptomyces* 16S rRNA gene tree is unstable.

**Fig. 2.**
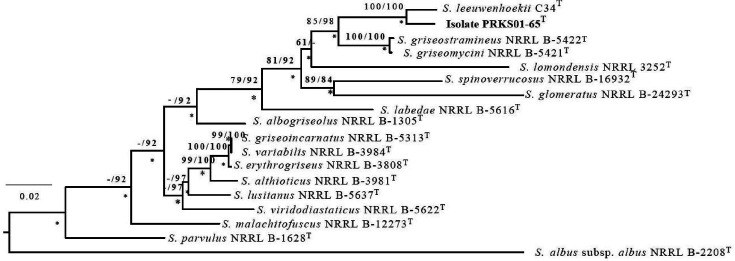
Maximum-likelihood tree based on concatenated partial sequences of five housekeeping genes (2528 nucleotides) showing relationships between isolate PRKS01-65^T^ and its closest phylogenetic neighbours. The tree was inferred using the GTR+GAMMA model. The branches are scaled in terms of the expected numbers of substitutions per site. The numbers above the branches are bootstrap support values of 60 % or over from the maximum-likelihood (left) and maximum-parsimony (right) analyses. Asterisks indicate branches of the tree that were found using the neighbour-joining tree making-algorithm. Bar, 0.02 substitutions per nucleotide position. The root of the tree was established using *Streptomyces albus* subsp. *albus* NRRL B-2208^T^.

Multilocus sequence analyses (MLSA) were undertaken based on 2528 nucleotides of partial sequences of five concatenated housekeeping genes: *atpD* (encodes ATP synthase F1 B-subunit), *gyrB* (gyrase B subunit), *recA* (recombinase protein A), *rpoB* (DNA-directed RNA polymerase B subunit) and *trpB* (tryptophan synthase B subunit). The resultant MLSA tree (Fig. 2) was based on information taken from the draft genome of the isolate and from available corresponding partial gene sequences of *Streptomyces* strains accessed from the NCBI GenBank database, the sequence data are presented in Table S2. Pairwise sequence similarities between the datasets were calculated using the GGDC web server [41] and phylogenetic analyses conducted with the GGDC webserver and the DSMZ phylogenomic pipeline [51]. In turn, multiple sequence alignments were generated using muscle software [40] and a maximum-likelihood tree inferred from alignment with RAxML [52] using rapid bootstrapping and the auto maximum-relative-error criterion [53]. In addition, a maximum-parsimony tree was inferred from the alignments with the Tree Analysis New Technology (TNT) program [54] using 1000 bootstraps with tree-bisection-and-reconnection branch swapping and 10 random sequence replicates. The sequences were checked for computational bias using the X2 test, as implemented in PAUP* (Phylogenetic Analysis Using Parsimony) [55].

It is apparent from the concatenated tree (Fig. 2) that most of the branches are supported by high bootstrap values providing further evidence that *Streptomyces* phylogenies based on MLSA gene sequences give better resolution than corresponding 16S rRNA gene trees [23, 56]. The MLSA tree underlines the close relationship between the isolate and *S. leeuwenhoekii* C34^T^ and shows that these strains are part to a well-defined clade that includes the type strains of *Streptomyces griceomycini* [57, 58], *Streptomyces griseostramineus* [57, 58] and *Streptomyces lomondensis* [59]., 60 the type strains of *S. glomeratus* [49], *S. labedae* [60] and *Streptomyces spinoverrucosus* [61] are located towards the periphery of this group. All the strains assigned to this clade produce spiral chains of spiny ornamental spores with the exception of isolate PRKS01-65^T^ and *S. leeuwenhoekii* C34^T^ [14], which form spiral chains of spores with smooth surfaces. The MLSA evolutionary distances between the isolates ranged from 0.016 to 0.141 (Table S3),; that is, well above the species level threshold of ≤0.007 used to distinguish between closely related species [56, 62]. In the present analyses, the *S. leeuwenhoekii* strain was found to be related to a markedly different set of *Streptomyces* type strains when compared with corresponding MLSA data reported by Busarakam *et al*. [14] who found that relationships between the *S. leeuwenhoekii* and the type strains of closely related *Streptomyces* species varied depending on the treeing algorithm used. However, greater confidence can be placed in the results of the present analysis since most of the branches in the tree are supported by high bootstrap values.

**Fig. 3.**
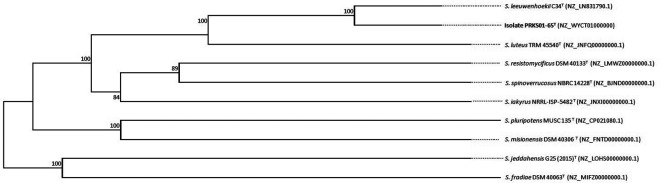
Phylogenomic tree showing relationships between isolate PRKS01-65^T^ and the most closely related *Streptomyces* type strains obtained using the TYGS platform. The number at the nodes are GBDP pseudo-bootstrap support values based on 100 replicates. The average branch support is 96.1 %. The tree is rooted at the midpoint. The accession numbers of genome sequences are given in parentheses.

A phylogenomic tree was generated based on whole-genome sequences of the isolate and its closest phylogenetic neighbors using the Type (Strain) Genome Server (TYGS) [63] available at http://tygs.dsmz.de. The minimum-evolutionary tree was inferred using FastME 2.1.6.1 software [64] based on the Genome blast Distance Phylogeny (GBDP). Distances were calculated from pairwise genome comparisons using formula d5 [41]. GBDP pseudo-bootstrap support values were calculated using 100 replicates and the tree rooted at the midpoint [65]. The phylogenomic tree (Fig. 3) confirms the close relationship between the isolate and *S. leeuwenhoekii* C34^T^ as these strains form a well-supported branch in the phlogenomic tree that is sharply separated from corresponding branches composed of the type strains of the most closely related *Streptomyces* species.

## Genomic characterization

The genome sequence of isolate PRKS01-65^T^ was compared with that of *S. leeuwenhoekii* C34^T^ (GenBank accession number AZSD0000000). Orthologous average nucleotide identity (orthoANI) [66] and digital DNA–DNA hybridization (dDDH) [42] values were calculated using the ANI calculator tool from the EZBioCloud (www.ezbiocloud.net/tools/ani) and the GGDC webserver (http://ggdc.dsmz.de/ggdc), respectively. The genome of the isolate was annotated and assigned to different functional classes using the rastwebserver available at http://rast.nmpdr.org/

The dDDH similarities between the genome of the isolate and *S. leeuwenhoekii* C34^T^ was 56.0 %, a value well below the 70 % threshold used to assign strains to the same genomic species [67]; the corresponding pairwise orthoANI value of 94.3 % was just below the threshold used to distinguish between closely related prokaryotic species [68, 69]. The relative distribution of the different functional gene classes in the genome of the isolate (Fig. S6) is similar to those found in the genomes of *S. leeuwenhoekii* C34^T^, ‘* Streptomyces coelicolor *’ A3(2) [70] and ‘*Streptomyces lividans*’ 66 [71].

## Phenotypic traits

Isolate PRKS01-65^T^ and *S. leeuwenhoekii* C34^T^ were examined for a broad range of phenotypic properties known to be value in distinguishing between *Streptomyces* species [8, 30, 72, 73]. The enzymatic profiles of the strains were determined using API-ZYM strips (bioMérieux) and biochemical, degradative and physiological properties using media and methods taken from Williams *et al*. [73]. The ability of the strains to grow under different temperature and pH regimes and in the presence of various sodium chloride concentrations were recorded using ISP2 agar as the basal medium. pH values were achieved using phosphate buffers. All of these tests were carried out in duplicate using a standard inoculum equivalent to 5.0 on the McFarland scale [74].

Identical results were obtained between the duplicated cultures for all of the phenotypic tests, several of which were weighted to distinguish between the isolate and *S. leeuwenhoekii* C34^T^ (Table 2). Thus, the isolate, unlike the *S. leeuwenhoekii* strain, produced β-glucosidase, lipase (C14) and α-mannosidase, hydrolysed urea, and degraded chitin and xanthine. In contrast, only *S. leeuwenhoekii* C34^T^ formed alkaline phosphatase and esterase (C4), metabolized adenine, elastin, guanine, tributyrin and Tween 40, and grew above pH 7.5 and at 4 and 50 °C. Both strains produced acid phosphatase, α-chymotrypsin, cystine arylamidase, esterase lipase, β-galactosidase, α-acetyl-β-glucoaminidase, naphthol-AS-BI-phosphohydrolase, trypsin and valine arylamidase, hydrolysed allantoin and arbutin, degraded hypoxanthine, starch, uric acid and Tween 80, reduced nitrate, and were catalase-positive. Neither strain produced α-fucosidase, α-glucosidase or β-glucoronidase, hydrolysed aesculin, or degraded casein, keratin, xylan or Tweens 20 and 60.

**Table 2.** Phenotypic properties that distinguish *Streptomyces* isolate PRKS01-65^T^ from *S. leeuwenhoekii* C34^T^ +, Positive result; −, negative result.

Characteristics	Isolate	* Streptomyces leeuwenhoekii *
	PRKS01-65^T^	C34^T^
**Cultural characteristics on oatmeal agar**		
Aerial spore mass	Greyish-olive	Olivaceous grey-green
Substrate mycelium	Greyish-olive	Yellowish-white
Diffusible pigment	−	Yellowish
**API-ZYM tests**		
Alkaline phosphatase	−	+
Esterase (C4)	−	+
Lipase (C 14)	+	−
α-Mannosidase	+	−
**Biochemical test**		
Urea hydrolysis	+	−
**Degradation tests**		
Adenine	−	+
Chitin	+	−
Elastin	−	+
Guanine	−	+
Tributyrin	−	+
Tween 40	−	+
Xanthine	+	−
**Tolerance tests**		
pH range	5.5–7.5	6.0–11
Temperature range (°C)	10–45	4–50
Growth in presence of NaCl (%, w/v)	1–5	1–10

The presence of putative biosynthetic gene clusters (BGs) encoding for natural products were sought in the genome of the isolate using the antiSMASH 5.0 platform [75] available at http://antismash.secondarymetabolites.org. AntiSMASH predicts BGCs and prospective products based on the percentage of genes from the closest known BGCs showing significant blast hits to the genome under consideration. In the present study, the genome of the isolate, unlike that of the *S. leeuwenhoekii* C34^T^, was associated with the production of several known antibiotics albeit with low gene similarities (<70 %), as exemplified by actinoallolide A (30 %; [76]), enduracidin (6 %; [77]), rustimicin (6 %; [78]) and tautomycin (27 %; [79]). In contrast, only the genome of *S. leeuwenhoekii* C34^T^ [18] contains the BGCs that express for a new family of ansamycin-like compounds, the chaxamycins, which show potent antibacterial and moderate anticancer activity [80].

It can be concluded from the present study that isolate PRKS01-65^T^ is a *bone fide* member of the genus *Streptomyces *. The isolate is closely related to *S. leeuwenhoekii* C34^T^, but not especially close to other *Streptomyces* species. The isolate and the *S. leeuwenhoekii* strain can be distinguished by a wealth of genotypic and phenotypic data, notably by a low dDDH value. It is proposed that isolate PRKS01-65^T^ (=NCIMB 15211^T^=CCMM B1302^T^=ICEBB-03^T^) be recognized as the type and only strain belonging to *Streptomyces harenosi* sp. nov. The isolate has a large genome (8 Mb) which contains putatively novel BGCs and hence can be considered gifted in the sense of Baltz [17].

## Description of *Streptomyces harenosi* sp. nov.

*Streptomyces harenosi* (ha.re.no’si. L. gen. n. *harenosi*, of a sandy place referring to the source of the organism).

Aerobic, Gram-stain positive, catalase-positive actinobacterium which forms an extensively branched substrate mycelium that bears aerial hyphae which differentiate into spiral chains of spores (0.8×1.0 µm) with smooth surfaces on yeast extract–malt extract agar. Brown diffusible pigments are formed on tryptone–yeast extract, yeast extract–malt extract and inorganic salts–starch agar. Grows from 10 to 45 °C, optimally at 28 °C, from pH 5.5 to 7.5, optimally at pH 7.0 and in the presence up to 5 % w/v NaCl. Allantoin, arbutin and urea are hydrolysed but not aesculin. Nitrate is reduced. Degrades chitin, hypoxanthine, starch, Tween 80, l-tyrosine, uric acid and xanthine, but not adenine, casein, elastin, keratin, Tween 20, tributyrin or xylan. Positive for acid phosphatase, α-chymotrypsin, cystine arylamidase, esterase lipase, β-galactosidase, α-acetyl-β-glucoaminidase, β-glucosidase, α-mannosidase, naphthol-AS-BI-phosphohydrolase, trypsin and valine arylamidase, but negative for α-fucosidase, α-glucosidase and β-glucoronidase. Whole-organism hydrolysates are rich in ll-A_2_pm, galactose and ribose, the predominant fatty acids are antesio-C_15 : 0_ and iso-C_16 : 0_, the major menaquinone is MK-9 (H8), and phosphatidylethanolamine is the diagnostic phospholipid. The DNA G+C content of the type strain is 73.36 mol% and the approximate genome size 8.0 Mb.

The type strain, PRKS01-65^T^ (=NCIMB 15211^T^=CCMM B1302^T^=ICEBB-03^T^), was isolated from a sandy soil sample collected from an arid sand dune at Parangkusumo, Yogyakarta Province, Java, Indonesia. The GenBank accession number of the assembled draft genome of strain PRKS01-65^T^ is WYCT00000000.

## Supplementary Data

Supplementary material 1
