# Supplementary material for: Streptomyces harenosi sp. nov., a home for a gifted strain isolated from Indonesian sand dune soil
Source: Int J Syst Evol Microbiol. 2020 Aug 21;70(9):4874–82. doi: 10.1099/ijsem.0.004346 (PMC7656270; doi:10.1099/ijsem.0.004346)
Supplement: Supplementary material 1 [file ijsem-70-4874-s001.pdf]

Supplementary materials

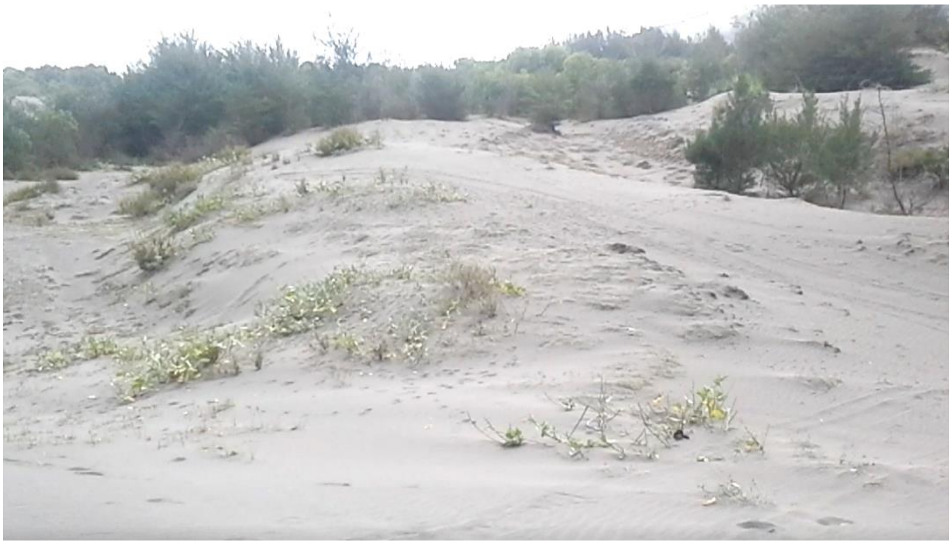

Fig. S1. Parangkusumo arid sand dunes, the source of isolate PRKS01-65^T

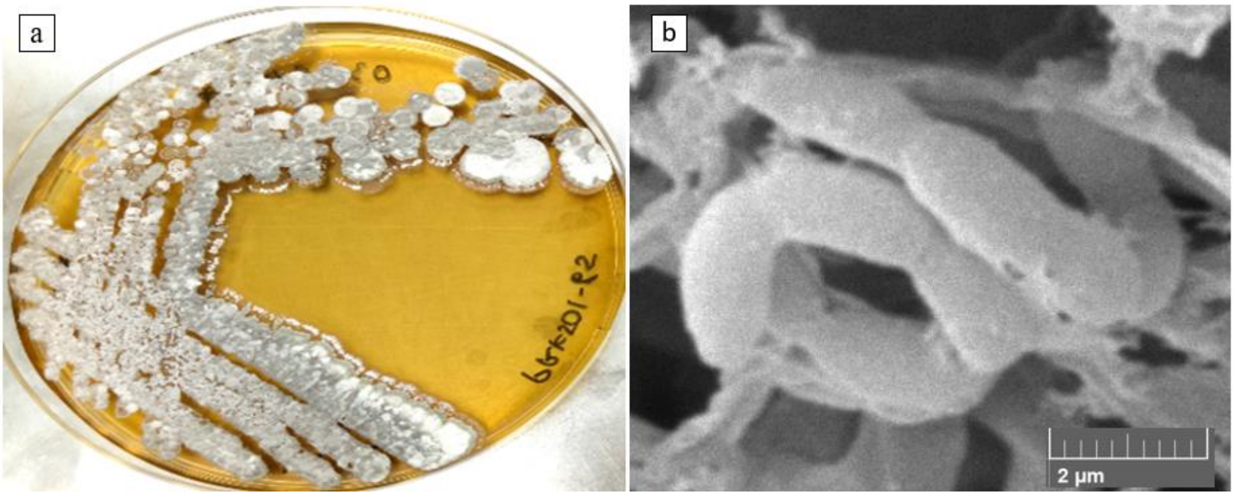

Fig. 2 Cultural and micromorphological properties of *Streptomyces* isolate PRKS01-65^T grown on ISP 2 agar plate for 14 days at 28°C. (a) Circular colonies with raised elevation, filamentous margins and a medium grey aerial spore mass (b) Scanning electron micrograph showing spiral chains of smooth surfaced spores. Bars: (b) 2 µm.

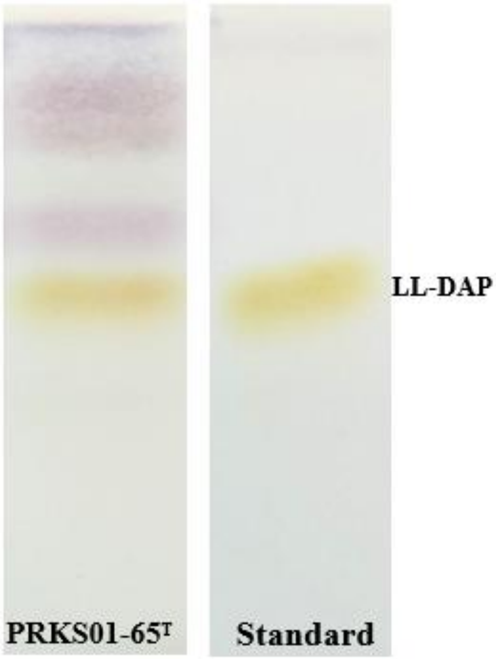

Fig. S3 Thin-layer chromatography of whole-cell hydrolysates showing that isolate PRKS01-65^{T} contains LL-diaminopimelic acid.

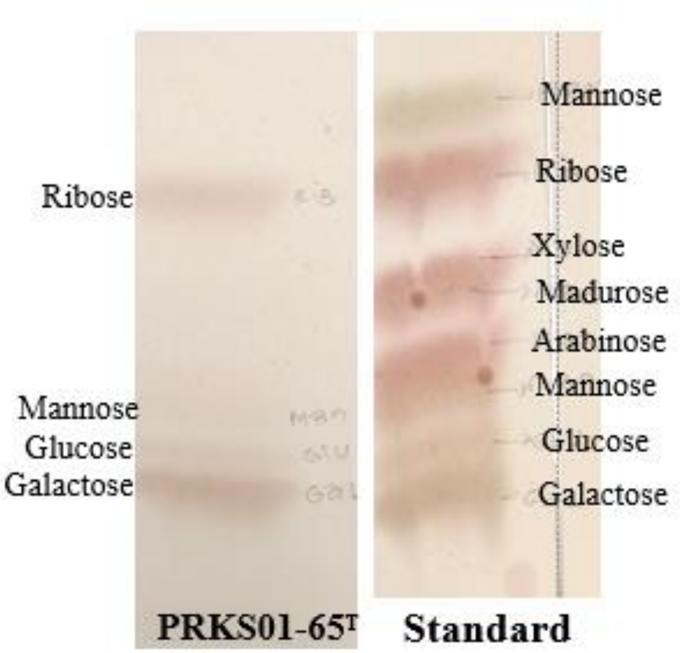

Fig. S4 Thin-layer chromatography of whole-cell hydrolysates showing that isolate PRKS01-65^{T} contains galactose, glucose, mannose and ribose.

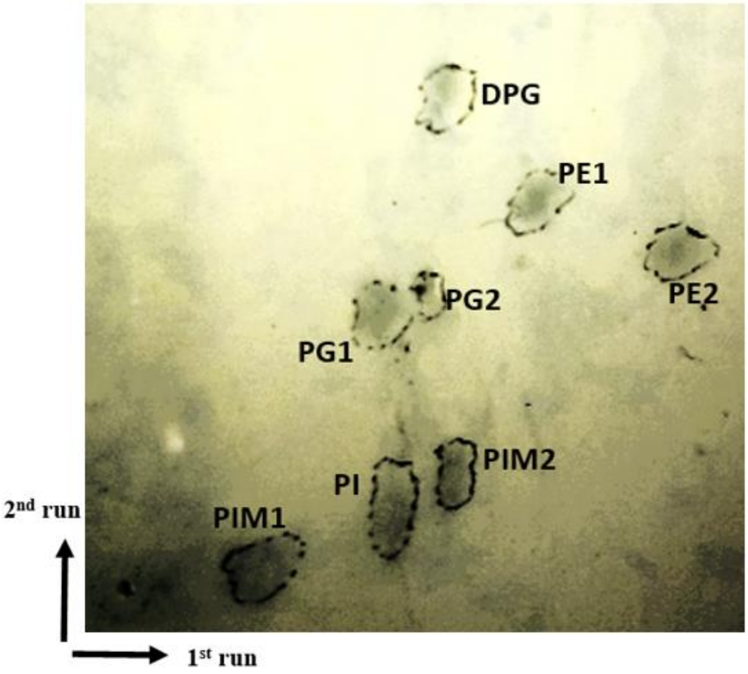

Fig. S5 Two-dimensional thin-layer chromatography of isolate PRKS01-65^T showing its polar lipid patterns following staining with molybdatophosphoric acid spray (Sigma P1518). Key: DPG, diphosphatidylglycerol; PG, phosphatidylglycerol; PE, phosphatidylethanolamine; PI, phosphatidylinositol and PIM, phosphatidylinositol mannosides. Solvent 1: chloroform: methanol: distilled water (65:25:4 v/v); Solvent 2: chloroform: glacial acetic acid: methanol: distilled water (80:12:15:4 v/v). The solvent 1 was used for the first chromatographic run where the TLC plate was set up in perpendicular position towards the solvent direction., whilst the solvent 2 was involved in second run where the position of the TLC plate and solvent are in the same direction.

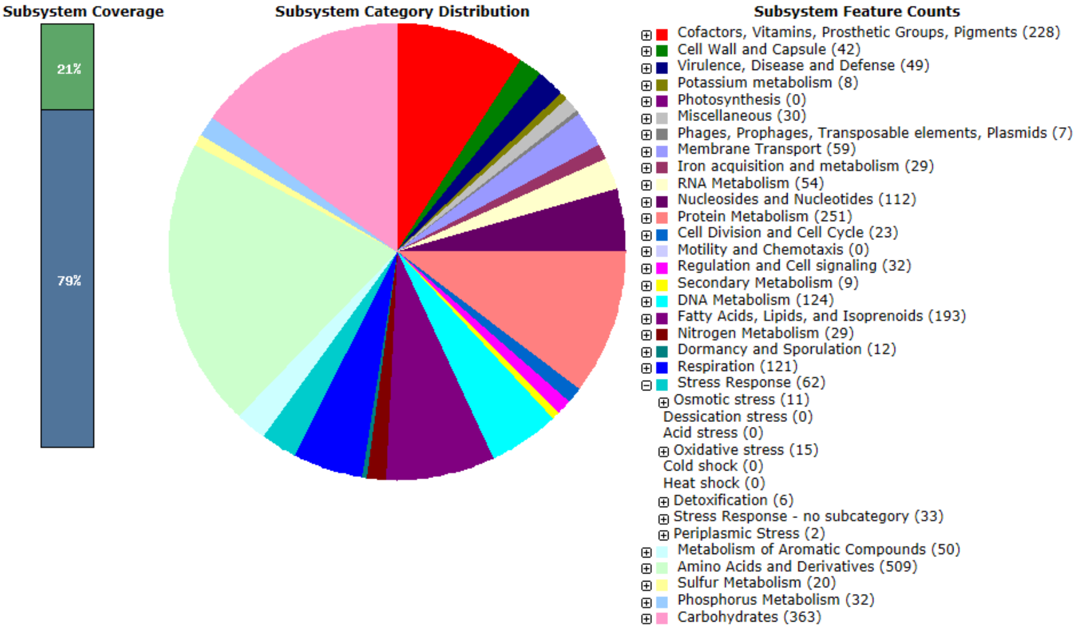

Fig. S6 Overview of *Streptomyces harenosi* PRKS01-65^T subsystem gene functions generated by analysis on the RAST-SEED webserver at http://rast.nmpdr.org/.

Table S1. Growth and cultural properties of *Streptomyes* isolate PRKS01-65^{T} and *S. leeuwenhoekii* C34^{T} on ISP media after 14 days at 28°C.

Characteristics	ISP media						
	1	2	3	4	5	6	7
Isolate PRKS01-65							
Growth	+++	+++	+++	+++	++	++	+++
Aerial spore mass	White-orangish	White-greyish	Greyish-olive	Soft brown	White	Pale yellow	White-greenish
Substrate mycelium	Medium brown	Medium brown	Greyish-olive	Soft brown	Soft brown	Pale yellow	Medium brown
Diffusible pigments	Dark brown	Soft brown	-	-	-	-	-
S. leeuwenhoekii C34^{T}							
Growth	++	+++	+++	++	++	++	++
Aerial spore mass	Olivaceous grey-green	Grey - yellowish green	Olivaceous grey-green	Olivaceous grey-green	Olivaceous grey-green	Olivaceous grey-green	Olivaceous grey-green
Substrate mycelium	Yellowish-white	Grey-yellow	Yellowish-white	Yellowish-white	Yellowish-white	Yellowish-white	Yellowish-white
Diffusible pigments	-	Pale yellow	Yellowish	Yellowish	-	Grey-yellow	Yellowish

+++, abundant growth; ++ very good growth.

Table. S2. Strains used in this MLSA study and -associated GenBank accession numbers.

Strain	Housekeeping genes				
	atpD	***gyrB***	***recA***	***rpoB***	***trpB***
**Isolate PRKS01-65^{T}**	MN938914	MN938915	MN938916	MN938917	MN938918
S. leeuwenhoekii C34^{T}	KJ137029	KJ137046	KJ137063	KJ137080	KJ137097
S. glomeratus NRRL B-24293^{T}	KT384569	KT384918	KT385267	KT388888	KT389238
S. griseoincarnatus NRRL B-5313^{T}	KT384580	KT384929	KT385278	KT388899	KT389249
S. erythrogriseus NRRL B-3808^{T}	KT384542	KT384891	KT385240	KT388861	KT389211
S. variabilis NRRL B-3984^{T}	KT384743	KT385091	KT385445	KT389063	KT389412
S. parvulus NRRL B-1628^{T}	KJ196367	KJ196369	KJ196371	KJ196373	KJ196375
S. labedae NRRL B-5616^{T}	KT384613	KT384962	KT385312	KT388933	KT389282
S. lusitanus NRRL B-5637^{T}	KJ196366	KJ196368	KJ196370	KJ996799	KJ196374
S. griseomycini NRRL B-5421^{T}	KJ137027	KJ137044	KJ137061	KJ996781	KJ137095
S. albogriseolus NRRL B-1305^{T}	KT384453	KT384802	KT385150	KT388772	KT389122
S. viridodiastaticus NRRL B-5622^{T}	KT384757	KT385105	KT385459	KT389077	KT389426
S. spinoverrucosus NRRL B-16932^{T}	KT384725	KT385074	KT385426	KT844525	KT389394
S. malachitofuscus NRRL B-12273^{T}	KT384634	KT384983	KT385334	KT388954	KT389303
S. althioticus NRRL B-3981^{T}	KT384460	KT384809	KT385157	KT388779	KT389129
S. griseostramineus NRRL B-5422^{T}	KT384585	KT384934	KT385283	KT388905	KT389254
S. lomondensis NRRL 3252^{T}	KT384626	KT384975	KT385326	KT388946	KT389295
S. albus subsp. *albus* NRRL B-2208^{T}	KF528055	KF528056	KF528057	KJ996610	KF528059

Table S3. MLSA distances between isolate PRKS01-65^T and its closest phylogenetic neighbours.

	**Strain**	**MLSA (Kimura two-parameter) distance**																
	**1**	**2**	**3**	**4**	**5**	**6**	**7**	**8**	**9**	**10**	**11**	**12**	**13**	**14**	**15**	**16**	**17**	**18**
1	**Isolate PRKS01-65^T**	**-**																
2	*S. leeuwenhoekii* C34^T	**0.016**																
3	*S. glomeratus* NRRL B-24293^T	**0.072**	0.074															
4	*S. griseoincarnatus* NRRL B-5313^T	**0.068**	0.069	0.088														
5	*S. erythrogriseus* NRRL B-3808^T	**0.067**	0.069	0.088	0.002													
6	*S. variabilis* NRRL B-3984^T	**0.068**	0.069	0.088	0.000	0.002												
7	*S. parvulus* NRRL B-1628^T	**0.069**	0.071	0.092	0.064	0.064	0.064											
8	*S. labedae* NRRL B-5616^T	**0.063**	0.062	0.078	0.051	0.051	0.051	0.072										
9	*S. lusitanus* NRRL B-5637^T	**0.060**	0.062	0.091	0.024	0.024	0.024	0.059	0.065									
10	*S. griseomycini* NRRL B-5421^T	**0.040**	0.041	0.070	0.062	0.062	0.062	0.064	0.058	0.058								
11	*S. albogriseolus* NRRL B-1305^T	**0.061**	0.060	0.088	0.037	0.037	0.037	0.063	0.054	0.038	0.055							
12	*S. viridodiastaticus* NRRL B-5622^T	**0.075**	0.076	0.102	0.031	0.031	0.031	0.066	0.072	0.031	0.072	0.041						
13	*S. spinoverrucosus* NRRL B-16932^T	**0.061**	0.062	0.071	0.075	0.075	0.075	0.076	0.067	0.072	0.054	0.068	0.087					
14	*S. malachitofuscus* NRRL B-12273^T	**0.065**	0.069	0.089	0.046	0.046	0.046	0.061	0.070	0.039	0.060	0.053	0.052	0.075				
15	*S. althioticus* NRRL B-3981^T	**0.066**	0.070	0.092	0.014	0.014	0.014	0.064	0.059	0.023	0.062	0.039	0.034	0.075	0.044			
16	*S. griseostramineus* NRRL B-5422^T	**0.041**	0.041	0.071	0.062	0.062	0.062	0.066	0.059	0.058	0.002	0.056	0.073	0.054	0.061	0.062		
17	*S. lomondensis* NRRL 3252^T	**0.071**	0.070	0.088	0.088	0.088	0.088	0.088	0.080	0.081	0.065	0.081	0.097	0.074	0.082	0.089	0.065	
18	*S. albus* subsp. *albus* NRRL B-2208^T	**0.138**	0.141	0.135	0.125	0.125	0.125	0.122	0.132	0.127	0.133	0.133	0.126	0.134	0.133	0.126	0.133	0.135
